# Parenterally Administered Porcine Epidemic Diarrhea Virus-Like Particle-Based Vaccine Formulated with CCL25/28 Chemokines Induces Systemic and Mucosal Immune Protectivity in Pigs

**DOI:** 10.3390/v12101122

**Published:** 2020-10-02

**Authors:** Chin-Wei Hsu, Ming-Hao Chang, Hui-Wen Chang, Tzong-Yuan Wu, Yen-Chen Chang

**Affiliations:** 1 Graduate Institute of Molecular and Comparative Pathobiology, School of Veterinary Medicine, National Taiwan University, Taipei 106, Taiwan; robby951159@gmail.com (C.-W.H.); huiwenchang@ntu.edu.tw (H.-W.C.); 2 Department of Bioscience Technology, Chung Yuan Christian University, Taoyuan 320, Taiwan; jhon2003111333@gmail.com; 3 Department of Medical Research, China Medical University Hospital, China Medical University, Taichung 406, Taiwan

**Keywords:** porcine epidemic diarrhea virus, VLP, chemokines, pig, vaccine

## Abstract

Generation of a safe, economical, and effective vaccine capable of inducing mucosal immunity is critical for the development of vaccines against enteric viral diseases. In the current study, virus-like particles (VLPs) containing the spike (S), membrane (M), and envelope (E) structural proteins of porcine epidemic diarrhea virus (PEDV) expressed by the novel polycistronic baculovirus expression vector were generated. The immunogenicity and protective efficacy of the PEDV VLPs formulated with or without mucosal adjuvants of CCL25 and CCL28 (CCL25/28) were evaluated in post-weaning pigs. While pigs intramuscularly immunized with VLPs alone were capable of eliciting systemic anti-PEDV S-specific IgG and cellular immunity, co-administration of PEDV VLPs with CCL25/28 could further modulate the immune responses by enhancing systemic anti-PEDV S-specific IgG, mucosal IgA, and cellular immunity. Upon challenge with PEDV, both VLP-immunized groups showed milder clinical signs with reduced fecal viral shedding as compared to the control group. Furthermore, pigs immunized with VLPs adjuvanted with CCL25/28 showed superior immune protection against PEDV. Our results suggest that VLPs formulated with CCL25/28 may serve as a potential PEDV vaccine candidate and the same strategy may serve as a platform for the development of other enteric viral vaccines.

## 1. Introduction

Porcine epidemic diarrhea (PED), caused by the porcine epidemic diarrhea virus (PEDV), is a contagious enteric viral disease that occurs specifically in pigs. PEDV is classified in the order *Nidovirales*, family *Coronaviridae*, and genus *Alphacoronavirus*. It contains a positive-sense, single-stranded, ~28-kilobase RNA genome, incorporated with the nucleocapsid (N) protein and enveloped in a membranous outer coat comprising three structural proteins: membrane (M), envelope (E), and S (S) proteins [1]. The S protein is a multifunctional molecular apparatus responsible for specific host and tissue recognition and induction of protective humoral as well as cellular immunities for viral neutralization and elimination [2,3,4]. The M protein, the most abundant structural protein, is responsible for the generation of virus particles. The small E protein, which accounts for a minority of the envelope component, plays a critical role in the viral morphogenesis and the final step of the budding process [5,6]. PEDV has a tropism for enterocytes of villous tips and further leads to atrophic enteritis [7,8]. Pigs of all ages can be affected by the disease; however, the severity of the gastrointestinal clinical signs is age-dependent as a result of the slower turnover of enterocytes and incomplete innate immunity in neonatal piglets than in post-weaning pigs [9,10]. Historically, PED has had minimal effect on piglets in a sporadic to epidemic manner. However, since 2010, PEDV has evolved into highly virulent viruses, designated as genogroup 2 (G2) strains, which are different from previous G1 strains and result in high mortality in neonatal piglets and devastating global outbreaks [11,12]. The high death rates cause tremendous economic losses, thus highlighting the requirement for an effective vaccine strategy.

In the past, the available tools for the prevention of PEDV infection have included live-attenuated or inactivated vaccines derived from G1 PEDVs, such as strains CV777, DR13, P5-V, and SM98-1 [13,14]. The traditional vaccines only confer partial cross-protection against the novel highly virulent PEDV G2 strains despite only up to 10% difference in the amino acid sequence of the S proteins between the G1 and G2 strains [1,15]. To control the outbreaks of virulent G2 viruses, two conditionally licensed vaccines have been commercialized in the United States. One is a recombinant vaccine expressing the PEDV S protein using a replication-deficient Venezuelan equine encephalitis virus packaging system, while the other is an inactivated vaccine based on the whole virus of non-S INDEL PEDV strain. The third vaccine candidate, which is in commercial development by Vaccine and Infectious Disease Organization—International Vaccine Centre, is a subunit vaccine that uses mammalian HEK-293 T cell-expressed PEDV S1 proteins [16]. However, the efficacy of these commercial vaccines in stimulating solid lactogenic immunity against the disease in suckling piglets has been inconsistent [17,18]. Many other attempts have also been made to develop effective vaccines, but most of them have been incapable of inducing mucosal immunity [19,20,21,22]. Although the immunogenicity of the live-attenuated as well as inactivated vaccines is considered more effective [23], the live-attenuated approach has safety concerns and poses risks of genetic recombination or virulence restoration of the wild-type strains. Besides, both the approaches are time-consuming in vaccine development [24,25]. In the quest for novel strategies, the next-generation vaccine is expected to promptly deal with RNA viruses harboring a high mutation rate [1]. The subunit approach is considered to be a better strategy for the development of a safer vaccine; however, it falls short on the ability to elicit the optimal immunogenicity. With developments in biotechnology, the use of virus-like particles (VLPs) as an advanced subunit vaccine offers advantages of being less time-consuming to develop, being safer, and eliciting adequate immunogenicity. Thus, it is a new balanced approach that avoids the trade-offs between security and immunogenicity with regard to vaccine development to the maximum extent.

Virus-like particles, which exhibit the size and geometry of the viral structures and closely resemble the corresponding native virion but without the viral genomic nucleic acids [26], have been shown to improve the immunogenicity. The absence of the genetic material renders VLPs replication-incompetent. The nanometer dimensions in a range of supramolecular particulate antigens (20–200 nm) allow VLPs to not only freely drain into lymph nodes but also to be efficiently uptaken by antigen-presenting cells [27,28], which is conducive to T cell responses, including CD4^+^ T helper cells and CD8^+^ cytotoxic T cells [29]. The efficient cross-presentation is mainly mediated by the appropriate size of the VLPs to activate lymphoid dendritic cells, which only reside in lymph nodes and are essential to cellular immune responses [30,31,32]. Additionally, VLPs characterized by nanoparticles with a highly repetitive array of conformational epitopes can directly interact with B cells and facilitate the subsequent humoral immunity [26,33,34]. Therefore, VLP is an effective and safe tool to stimulate both cellular and humoral immunity in the absence of intracellular replication. Considering the high complexity of the enveloped PEDV VLPs, a polycistronic baculovirus expression vector system (P-BEVS), which involves co-expressing polycistronic genes via internal ribosome entry sites, has been adopted to produce VLPs comprising all of the envelope components [35,36]. Although a recent report has demonstrated that BEVS can successfully produce PEDV VLPs that are capable of inducing PEDV-specific humoral immunity in mice [37], the efficacy of PEDV VLPs against PEDV challenge in pigs still needs further evaluations.

Majorly targeting the superficial villous enterocytes, the PEDV is categorized as a type I enteropathogenic virus, and local mucosal and cellular immunities are important to control the viral infection [38]. The establishment of mucosal immunity requires a specific microenvironment to promote the development of the defense models, such as immunoglobulin class switching to IgA and J chain, and program surface homing ligands or receptors of immune cells [39]. Therefore, in the natural situation, the best immunization route is through the affected compartment. Many studies have proved that oral inoculation of virulent enteric viruses had better induction of mucosal IgA [35,40,41]. However, oral administration of equal vaccine dosage is technically difficult and labor-intensive in the swine industry. Intramuscular injection is a common immunization route in the field due to its operational practicability, but at the expense of protective mucosal immunity. To enhance the mucosal immune responses of parenteral administration, studies have used chemokines as molecular adjuvants to potentially drive the immune responses toward intestinal mucosal immunity. Among the various chemokines, small and large intestine-associated chemokines, CCL25/TECK (thymus-expressed chemokine) and CCL28 (mucosae-associated epithelial chemokine) [36], have been shown to up-regulate the localization of immune cells with CCR9 and CCR10, respectively, to the mucosal sites after systemic immunization [42,43,44]. Besides, the synergistic effects of CCL25 and CCL28 in trafficking IgA^+^ cells into the intestines has been confirmed in mice [45]. Our previous work also proved that inactivated PEDV co-adjuvanted with porcine CCL25 and CCL28 is competent in enhancing mucosal and systemic antibody responses and protective efficacy in pigs [46]. Therefore, intramuscular injection of an immunogen in combination with CCL25 and CCL28 could be a potential strategy for developing vaccines against enteric diseases.

In the present study, a P-BEVS-derived PEDV VLP comprising the S, M, and E proteins of the highly virulent PEDV G2 strain was successfully generated. The immunogenicity of the VLP, including systemic PEDV S-specific IgG, mucosal IgA, and cellular immunity, was evaluated in a 4-week-old pig model, and accompanied with an assessment of the protection against a homogenous PEDV challenge at 11-week-old pigs. Furthermore, an advanced statistical method known as generalized estimating equations (GEE), which has high statistical power in a small sample size as well as the ability to examine the effects of multiple factors on an outcome, was used for data analysis.

## 2. Materials and Methods

### 2.1. Plasmid Construction

**Figure 1:**
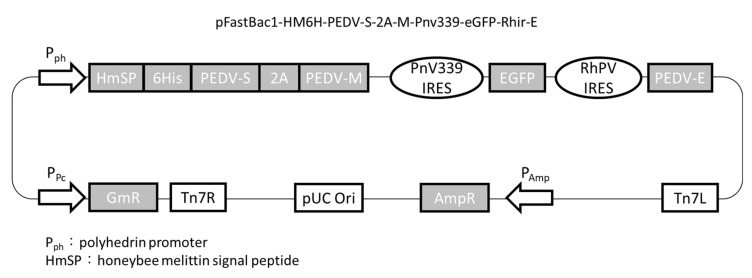
The construction map of the plasmid pFastBac1- HM6H- PEDV- S- 2A- M- Pnv339- eGFP- Rhir-E. The recombinant Taiwan G2b PEDV-PT strain spike (S) gene, with the honey bee melittin signal peptide and 6xHis-tag, and the membrane (M) gene linked by the 2A-like sequence were driven by the polyhedrin promoter. The envelope (E) gene was translated through the internal ribosome entry site (IRES) of Rhopalosiphum padi virus (RhPV). Enhanced green fluorescent protein (EGFP) gene was inserted into the plasmid and expressed by a truncated perina nuda picorna-like virus IRES (PnV339 IRES).

The S nucleotide sequence with the original signal peptide replaced by the honeybee melittin signal peptide was kindly provided by Dr. Yu-Chan Chao at Academia Sinica. The S, M, and E genes were derived from the Taiwan G2b PEDV-PT strain (Genbank accession no. KP276252). The S gene was codon-optimized to an insect cell system and synthesized by ProTech (ProTech, Taipei, Taiwan). The 2A-like sequence isolated from *Perina nuda* virus (PnV) and the M and E genes were inserted into the XbaI and NotI sites, respectively, in the pBac-mcsI-PnV339-eGFP-Rhir-mcsII vector [47]. Following that, the 2A-M-PnV339-eGFP-Rhir-E sequence, along with the honeybee melittin signal peptide, hexahistidine tag, and S gene, was included in the pFastBac1 plasmid (Invitrogen, Carlsbad, CA, USA) using the NEBuilder^®^ HiFi DNA Assembly Kit (New England Biolabs, Ipswich, MA, USA) to generate pFastBac1-HM6H-PEDV-S-2A-M-PnV339-eGFP-Rhir-E (Figure 1). It was used as the recombinant baculovirus transfer vector to recombine with the bacmid DNA in *E. coli* (strain DH10Bac, Invitrogen). The recombinant bacmid containing PEDV S, M, and E genes was transfected into Sf21 cells using Cellfectin^TM^ (Life Technologies, Carlsbad, CA, USA) to generate the recombinant baculovirus, SME-Bac.

### 2.2. Generation of VLPs

Sf21 cells were passaged to reach a cell density of 1 × 10^7^ in each T75 flask. After SME-Bac infection at a multiplicity of infection (MOI) of 1 in Sf21 cells for 5 days, the culture medium was collected, centrifuged at 600× *g* for 5 min to remove the cell debris, and passed through a 0.22 µm filter. VLPs in 10 mL of the supernatant were collected using sucrose cushion ultracentrifugation at 9000× *g*, 4 °C, for 90 min using a Beckman SW-41 rotor (Beckman Instruments, Spinco Division, Palo Alto, CA, USA). The precipitated VLPs were resuspended in 100 µL phosphate-buffered saline (PBS).

### 2.3. Indirect Fluorescent Antibody Test

Sf21 cells were passaged to reach a cell density of 2 × 10^5^ cells/well of a 24-well plate. After the Sf21 cells were infected with 5 MOI of SME-Bac for 4 days, each well was washed three times with 200 µL of PBS supplemented with 0.1% tween-20 (PBST) and fixed using 200 µL of 4% paraformaldehyde on ice for 20 min. The paraformaldehyde was then removed and each well was blocked with 200 µL blocking buffer (3% bovine serum albumin) and incubated at room temperature (RT) for 1 h. Two hundred microliters of anti-PEDV S monoclonal antibody, P4B [48], diluted in blocking buffer (1:200 ratio) was added to each well and allowed to incubate at RT for 2 h. After three PBST washes and a 1 h incubation with Alexa Flour^®^ 594-conjugated AffiniPure goat anti-mouse IgG (Jackson ImmunoResearch, West Grove, PA, USA) diluted 1:400 in blocking buffer, the wells were washed three times with PBST and observed under a fluorescence microscope.

### 2.4. Western Blotting

The precipitated VLPs were loaded onto an 8% sodium dodecyl sulfate-polyacrylamide electrophoresis gel. After electrophoresis, the proteins were transferred to a methanol-activated polyvinylidene fluoride membrane at 300 mA for 180 min. The recombinant S protein was detected using a rabbit anti-His-tag polyclonal antibody (1:2000 dilution, Rockland, NY, USA). Following this, a horseradish peroxidase (HRP)-conjugated goat anti-rabbit IgG (1:5000 dilution, Cell Signaling Technology, Massachusetts, USA) was used as the secondary antibody for signal detection. The membrane was developed using Immobilon^TM^ Western ECL Substrate (Millipore, MA, USA).

To determine the expression of S protein on the VLPs, the PEDV-challenged and PEDV-free porcine sera were used to perform Western blot. The prepared VLPs and EGFP-Bac, which was the same plasmid without PEDV S, E, and M sequences (pFastBac1-HM6H-P-2A-PnV339-eGFP-Rhir) and acted as a negative control, were loaded and protein electrophoresis was conducted. The following steps were similar to those described above, but instead, the primary and secondary antibodies were replaced by PEDV-challenged porcine serum (1:1000 dilution in blocking buffer) and HRP-conjugated goat anti-pig IgG (1:1000 dilution; Kirkegaard & Perry Laboratories, MD, USA), respectively.

### 2.5. Characterization of VLPs Using Electron Microscopy

For the preparation of the microscopic grids, an aliquot of 10 µL of samples was added to the carbon-coated grid for 1 min and then removed using a filter paper. The grids were stained with 2% phosphotungstic acid (PTA) for 1 min. Following that, the excess PTA was drained and the grids were completely dried for 6 h before being examined under a Tecnai G^2^ Spirit TWIN transmission electron microscope (FEI Company, OR, USA).

### 2.6. Expression and Purification of CC Chemokines

The CC chemokines, CCL25 and CCL28, were prepared as described in a previous study [46]. The aqueous formulations comprised the immunogen with/without CC chemokines for different groups (Table 1). The amounts of VLP and chemokines CCL25 or CCL28 were quantified using Western blot followed by ImageJ analysis and Pierce^TM^ BCA Protein Assay Kit (Thermo Fisher Scientific, Waltham, MA, USA).

### 2.7. Cell Lines and Viruses

The highly virulent viral stock of PEDV Pintung 52 passage 7 (PEDVPT-P7) was derived from PEDVPT-P5 (GenBank accession no. KY929405) as described in previous studies [19,20,21]. Vero C1008 cells (American Type Culture Collection no. CRL-1586) were used for viral preparation and the neutralizing assay. The culture medium was Dulbecco’s modified Eagle medium (DMEM, Gibco, Grand Island, NY, USA) supplemented with 10% fetal bovine serum (GE Healthcare, Uppsala, Sweden), 250 ng/mL amphotericin B, 100 U/mL penicillin, and 100 µg/mL streptomycin. The viral titer of PEDVPT-P7 was 1.78 × 10^5^ TCID_50_/mL, as determined using the endpoint titration assay with a ten-fold serial dilution in triplicates.

### 2.8. Immunization Program of Pigs

Twenty-three-week-old castrated male Large White × Duroc crossbred pigs, which were PEDV-seronegative and had no PEDV fecal shedding, were selected from a conventional pig farm. These pigs were randomly separated into three groups, including the VLP group (*n* = 7), VLP+CCL (*n* = 7), and control (*n* = 6) groups. After acclimation for one week, pigs in each group were intramuscularly primed with the 0.5 mL regimen shown in Table 1 on day 0. In the control group, pigs were immunized with adjuvanted Dulbecco’s PBS (DPBS, Gibco), containing Freund’s complete adjuvant (Sigma-Aldrich, St. Louis, MO, USA). Pigs in VLP and VLP+CCL groups were injected with 1.8 mg VLP (containing 0.2 µg S protein) diluted in 0.5 mL adjuvanted DPBS with or without 30 µg CCL25 and 30 µg CCL28. When boosting on days 14 and 35, the formulations were identical to those used for priming, except that the Freund’s complete adjuvant was replaced with Freund’s incomplete adjuvant (Sigma-Aldrich). At 0, 14, 28, and 49 days post-prime immunization (DPPI), blood anti-coagulated using ethylenediamine tetraacetic acid (EDTA) was collected along with oral swabs for detecting IFN-γ-producing cells, systemic IgG and neutralizing antibody titers, and mucosal IgA titers. At 49 DPPI, all the pigs were orally challenged with 5 mL of 10^5^ TCID_50_/mL PEDVPT-P7 to evaluate the protective efficacy. Stool consistency was monitored daily along with the collection of fecal swabs for detecting viral shedding. The animal experimental procedure was reviewed and approved by the Institutional Animal Care and Use Committee of the National Taiwan University (Taiwan, China) with the approval no. NTU107EL-00105.

### 2.9. Evaluation of Systemic IgG and Mucosal IgA Levels

PEDV-specific antibodies in the plasma and saliva were detected using an in-house PEDV S-based indirect enzyme-linked immunosorbent assay, as described in the previous study [48]. Briefly, the 96-well flat-bottom microplates (Thermo Fisher Scientific) were coated with 2 µg/mL recombinant S protein diluted in coating buffer (KPL, Gaithersburg, MD, USA) at 4 °C overnight. Following that, each well was washed six times with 200 µL/well of washing buffer (KPL) using a microplate washer (BioTek Instruments, Inc., Winooski, VT, USA) and then blocked with 300 µL/well of blocking buffer (KPL) at RT for 1 h. After six times of washing, the plasma IgG was evaluated by adding 100 µL per well of 40-fold diluted plasma samples in blocking buffer (KPL) and incubating at RT for 1 h, while the salivary IgA titer was detected by adding 100 µL per well of two-fold diluted salivary supernatant in blocking buffer (KPL) and incubating at 4 °C overnight. Following washing at the end of incubation, 100 µL/well of HRP-conjugated goat anti-pig IgG (KPL) at a 1:1000 dilution and HRP-conjugated goat anti-pig IgA (Abcam, Cambridge, UK) at a 1:5000 dilution were used to detect porcine IgG and IgA, respectively. After incubation at RT prior to the wash step, 50 µL of ABST^®^ Peroxidase Substrate System (KPL) was added to each well and the reaction was allowed to develop at RT for 5 and 45 min for IgG and IgA measurements, respectively. The reactions were stopped by adding 50 µL of stopping solution (KPL). The optical density (OD) values were read at 405 nm using the EMax^®^ Plus Microplate Reader (Molecular Devices, Crawley, UK). The IgG and IgA titers have been expressed as sample-to-positive ratios (S/P ratios), defined as the difference between the OD values of the sample and the negative control divided by the difference between the OD values of the positive and negative controls. The positive control samples were plasma or salivary samples from pigs challenged with PEDV in previous experiments.

### 2.10. Neutralizing Antibody Assay

For the evaluation of neutralizing antibody titers, 100 µL of Vero cells were seeded into 96-well culture plates (Thermo Fisher Scientific) at a density of 3 × 10^5^ cells/mL and incubated at 37 °C, 5% CO_2_ overnight to reach 80–90% confluency. Plasma samples of the pigs were heated at 56 °C for 30 min to inactivate the complement. The ten-fold diluted, inactivated plasma samples were two-fold serially diluted in post-inoculation (PI) medium containing DMEM supplemented with 0.3% tryptose phosphate broth (Sigma-Aldrich), 0.02% yeast extract (Acumedia, Lansing, CA, USA), and 10 µg/mL trypsin (Gibco). Each well contained 50 µL of 100 TCID_50_ PEDVPT-P5 and 50 µL of diluted plasma samples and was incubated at 37 °C, 5% CO_2_, for 1 h. Subsequently, the mixture was added to 90%-confluent Vero cells following two washes with PI medium and allowed to incubate at 37 °C, 5% CO_2_, for 1 h. The mixture was then removed and fresh PI medium was added and allowed to incubate at 37 °C, 5% CO_2_, for one day. The cytopathic effects were then observed under an inverted light microscope (Nikon, Tokyo, Japan). The neutralizing titer was defined as the last dilution without cytopathic effects.

### 2.11. Isolation of Peripheral Blood Mononuclear Cells

For the functional assay of peripheral blood mononuclear cells (PBMCs), 10 mL of blood, containing 1 mL of 1% EDTA (Merck, Darmstadt, Germany) at pH 7.5–8.0, was collected and centrifuged at 1811× *g*, 4 °C, for 30 min. The buffy coat was harvested and diluted in 6 mL of RPMI-1640 medium (Gibco) for subsequent density gradient centrifugation. The diluted buffy coat was gently applied to an equal volume of Ficoll-Paque^TM^ PLUS (GE Healthcare), and centrifuged at 1811× *g*, 20 °C, for 30 min. The isolated PBMCs, located at the interface of RPMI-1640 and Ficoll-Paque^TM^ PLUS (GE Healthcare), were collected and mixed with three volumes of sterile ammonium chloride potassium (ACK) lysis buffer, containing 0.15 M NH_4_Cl, 1.0 M KHCO_3_, and 0.01 M EDTA at pH 7.2–7.4. After incubation at 4 °C for 5 min, the cells were centrifuged at 201× *g*, 20 °C, for 10 min to collect the erythrocyte-free pellets. The pellet was resuspended in RPMI-1640 medium and centrifuged at 129× *g*, 20 °C, for 10 min to get rid of the platelets. The platelet-free pellets were then diluted to a final concentration of 3 × 10^6^ PBMCs/mL in CTL-Test^TM^ medium (Cellular Technology, LLC, Cleveland, OH, USA) for subsequent use.

### 2.12. Enzyme-Linked Immunospot Assay of PEDV S-Specific IFN-γ

According to the manufacturer’s instructions, the total PEDV S-specific IFN-γ secreting-cells were analyzed using enzyme-linked immunospot (ELISPOT) assay with anti-porcine IFN-γ pre-coated plates and detecting antibodies purchased from Cellular Technology. The freshly isolated PBMCs were seeded into the anti-porcine IFN-γ pre-coated plates at a density of 3 × 10^5^ cells/well and incubated at 37 °C for 24 h with CTL-Test^TM^ medium (mock) or CTL-Test^TM^ medium containing 10 µg/mL of in-house full-length recombinant S (treatment) [48] or 0.1 µg/mL concanavalin A (Sigma-Aldrich) (positive control). After incubation for one day, IFN-γ detection and color development were performed according to the manufacturer’s protocol. The scanning and counting were performed using CTL ImmunoSpot^®^ analyzers and the results were analyzed using ImmunoSpot^®^ software version 7.0.23.2.

### 2.13. Stool Consistency Scoring and Body Weight Measurement

The clinical signs for each pig were monitored and recorded daily. Based on previous studies [22,46], the severity of diarrhea was graded as 0: normal consistency; 1: loose consistency; 2: semi-fluid consistency; 3: liquid consistency. The body weight (BW) of each pig was measured weekly.

### 2.14. RNA Extraction, Complementary DNA Synthesis, and Probe-Based Quantitative Real-Time PCR

To detect fecal viral shedding after the viral challenge, feces collected from rectal swabs were resuspended in 900 µL of DPBS (Gibco) and mixed using a vortex. The resuspended samples were centrifuged at 13,793× *g* for 10 min. The viral RNA was extracted using the Cador^®^ Pathogen 96 QIAcube^®^ HT Kit according to the manufacturer’s instructions (Qiagen, Hilden, Germany). Reverse transcription was performed using the QuantiNova^TM^ Reverse Transcription Kit (Qiagen) to synthesize cDNA for subsequent quantitative real-time PCR, as described previously [49]. The detection limit of the assay was 4.7 log_10_ RNA copies per mL based on the standard curve of the in vitro transcribed PEDV RNA.

### 2.15. Statistical Analysis

The collected data were characterized by a small sample size, missing data, and independence between repeated measurements during the period of study with a non-normal distribution, as checked using the Shapiro–Wilk test. Traditional modeling techniques, such as repeated measures analysis of variance, reduce the statistical power and contribute to interpretation issues by list-wise deletion of missing data and data distortion after transformation [50,51]. The use of alternative statistical methods, such as GEE and linear mixed effects, for analysis of the longitudinal data overcomes the problems of valuable data reduction and inflexible correlation structure. Besides, several studies have proved that these advanced statistical methods are capable of enhancing power even with a small sample size and missing data as compared to the traditional models [52,53]. In the collected data set, GEE as an extension of the generalized linear model is preferable over linear mixed effects due to the robust standard errors, no limitation of normality, and more emphasis on the population-level trajectories rather than within-subject changes [54]. The aforementioned advantages render GEE increasingly popular in clinical trials.

A descriptive statistical analysis of all the experimental data was performed using a 95% confident interval for mean values to summarize the sample features. GEE with an exchangeable correlation structure and identity link function was used for further inferential statistics. The outcome variables (systemic IgG, oral IgA, neutralizing antibody titers, and fecal viral shedding) were modeled with a treatment factor (control; VLP; VLP+CCL), a repeated measure factor (pre-vaccination; 14, 28, and 49 DPPI), and a BW factor. Based on the significant interaction terms (group × BW; time × BW; group × time × BW), BW was considered as a covariate; therefore, the model was adjusted at a fixed BW before further statistical analysis. Given that the significant interaction term (group × time) was noted in all the data sets, the results were presented as post hoc comparisons of the simple main effects, which revealed the effect of the different treatments at different times. The data were analyzed using SPSS (SPSS for Mac, v. 24.0; IBM, Chicago, IL, USA). A *p* value of 0.05 was considered statistically significant. All the graphics were prepared using GraphPad Prism 6.0 (GraphPad software, San Diego, CA, USA). Results have been expressed as mean ± standard error of the mean (SEM).

## 3. Results

### 3.1. Preparation and Characterization of PEDV VLPs Expressed Using Recombinant Baculovirus, SME-Bac

**Figure 2:**
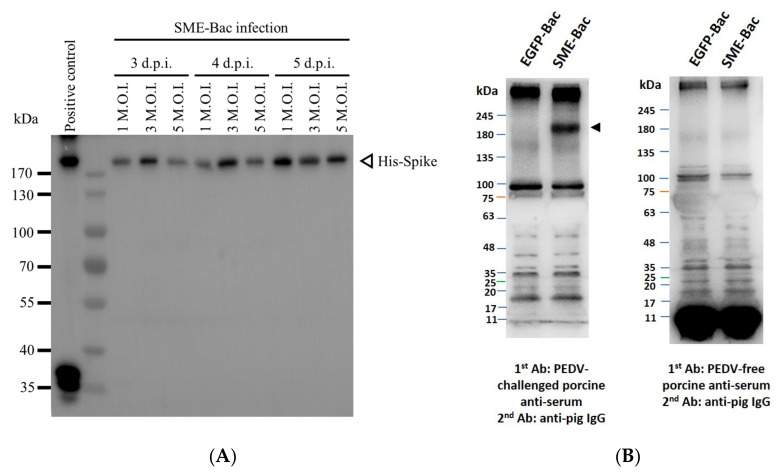
The detection of PEDV spike (S) proteins in the infected Sf21supernatant in various virus-like particles (VLP) production conditions by Western blot. (**A**) The samples were purified by conducting sucrose cushion and detected by anti-His tag antibodies. Positive control was Sf21 cells infected with S-Bac. (**B**) The Western blots of the VLPs stained with PEDV-challenged porcine serum were conducted. The protein bands of PEDV S protein are indicated by arrow heads. The EGFP-Bac was the sample collected from Sf21 cells infected with pFastBac1-HM6H-P-2A-PnV339-eGFP-Rhir and acted as a negative control.

**Figure 3:**
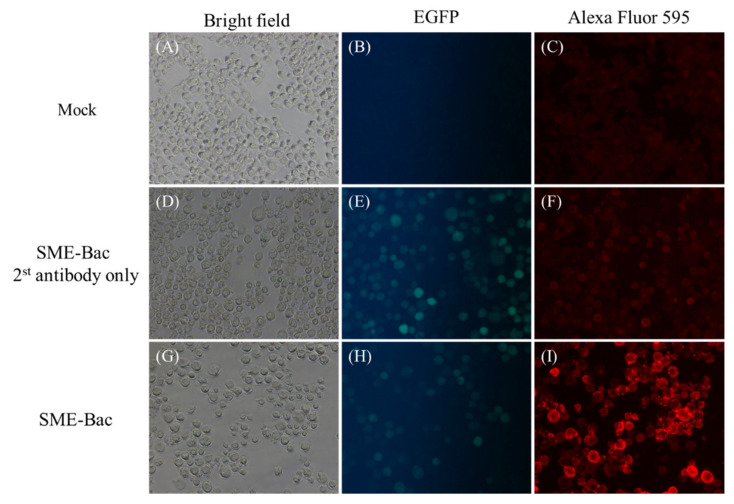
The detection of recombinant spike (S) proteins in SME-Bac infected cells. Sf21 cells in a total number of 2 × 10^5^ cells were infected with 5 MOI of SME-Bac for 4 days. (**A**,**D**,**G**) The morphologies of the Sf21 cells with different treatments under bright field. (**B**,**E**,**H**) The Sf21 cells successfully infected by SME-Bac showed green fluorescence under fluorescent microscope. (**C**,**F**,**I**) In the indirect fluorescent assay, Sf21 cells expressing PEDV S protein displayed red fluorescent signals under fluorescent microscope. The mock-infected cells and SME-Bac-infected cells stained with secondary antibody were used as negative controls.

After the transfer construct pFastBac1-HM6H-PEDV-S-2A-M-Pnv339-eGFP-Rhir-E in the recombinant baculovirus, SME-Bac was transduced into Sf21 cells, the recombinant S protein expression and VLP production were evaluated using Western blot. Using a previously characterized PEDV S-displaying baculovirus (S-Bac) [22] as the positive control, the His-tagged S protein was successfully detected in the culture medium. The amounts of S protein in the VLP-containing supernatants after three–five days of infection with 1, 3, and 5 MOI of SME-Bac are shown in Figure 2A. The S proteins were slightly larger than 170 kDa in size. The optimal VLP production condition was found to be five days after infection with 1 MOI of SME-Bac. To determine the recombinant S protein being displayed on the surface of the VLPs, the infected Sf21 cells were probed using an indirect immunofluorescence assay with the PEDV S monoclonal antibody and Alexa Flour^®^ 594-conjugated secondary antibody. Compared to the non-infected cells, there were strong fluorescence signals on the plasma membrane of Sf21 cells transduced with SME-Bac (Figure 3I), indicating that the S proteins were successfully being expressed on the surface of SME-Bac-infected Sf21 cells. Furthermore, to identify the S protein expressed on the VLPs, the Western blotting detected by PEDV-challenged porcine serum was performed. The result is demonstrated in Figure 2B. The size of protein bands pointed out by the arrow heads was approximately 200 kDa, indicative of the S protein.

**Figure 4:**
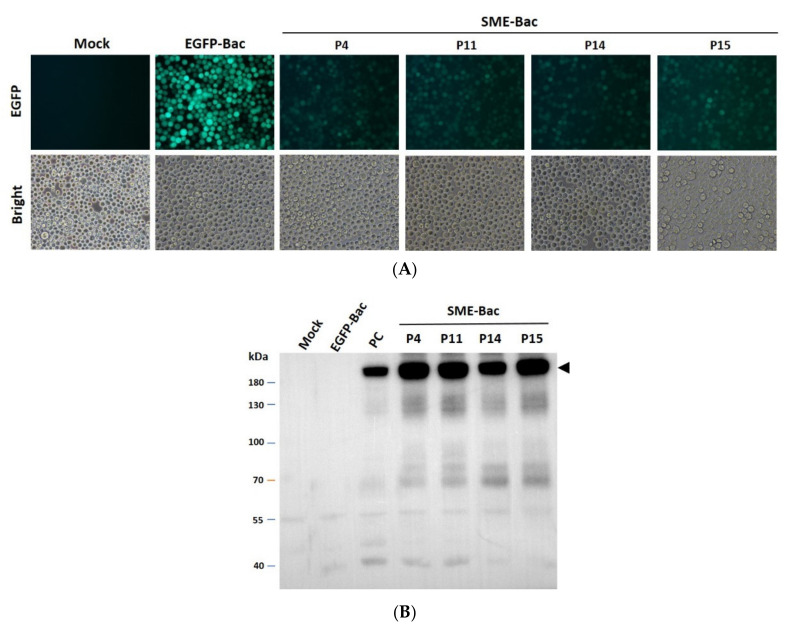
Detection of PEDV spike protein expression in Sf21 cells infected with different passages of SME-Bac. (**A**) The expression level of EGFP of Sf21 cells infected with 1 MOI of EGFP-Bac or SME-Bac passages 4, 11, 14, or 15 (P4, P11, P14, or P15) for 4 days were observed by fluorescence microscopy. (**B**) The PEDV S protein expression in the cell lysates after SME-Bac passages 4, 11, 14, or 15 infection was detected by Western blotting. The arrow head demonstrates the amount of S protein in different passages.

To evaluate the stability of P-BEVS that express PEDV VLP, we compared the infectivity and S protein expression level of passages 4, 11, 14, or 15 of SME-Bac. In the SME-Bac, EGFP, translated under the control of PnV339 IRES, could be used to monitor the infectivity of the recombinant baculovirus during VLP preparation after multiple passages. As shown in Figure 4A, the green fluorescence was similar between Sf21 cells infected with SME-Bac passages 4, 11, 14, and 15. However, the EGFP still can be translated via the cap-dependent mechanism when the S-2A-M sequences are lost during passaging. To further confirm the results, we monitored the S proteins expression in the passages 4, 11, 14, and 15. It showed that the expression level of S protein in both passages were consistent and similar (Figure 4B). Thus, the stability of SME-Bac could sustain at least 15 passages in the present study.

### 3.2. Negative Staining Electron Microscopy of PEDV VLPs

**Figure 5:**
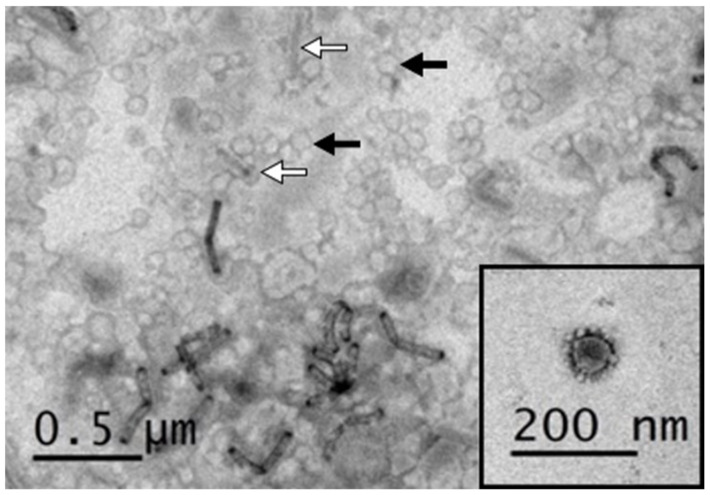
PEDV-like particles released in the culture supernatant of 1 MOI SME-Bac-infected Sf21 cells after 5-day infection. The electron micrograph demonstrates the morphology of VLPs (black arrows and inset figure) and baculovirus virions (white arrows).

To investigate whether the co-expressed S, M, and E proteins can successfully assemble into VLPs, the sample was collected and purified from the supernatant of SME-Bac-infected Sf21 cells, and then examined using transmission electron microscopy (TEM). The TEM image has been shown in Figure 5. There were numerous VLPs, approximately 100 nm in diameter, displaying similar morphology to coronavirus (black arrows and inset figure) and some rod-shaped virions, approximately 200 nm in size, resembling baculovirus (white arrows). Although a small number of baculoviruses were precipitated together with the VLPs, further purification of VLP for removing the baculovirus was not performed. Since baculovirus itself can elicit innate immune responses by regulating cytokines and promote B cell and T cell activation [55,56,57], the remaining baculoviruses are used as an adjuvant to enhance the effect of the VLPs.

### 3.3. Changes in Body Weight

**Figure 6:**
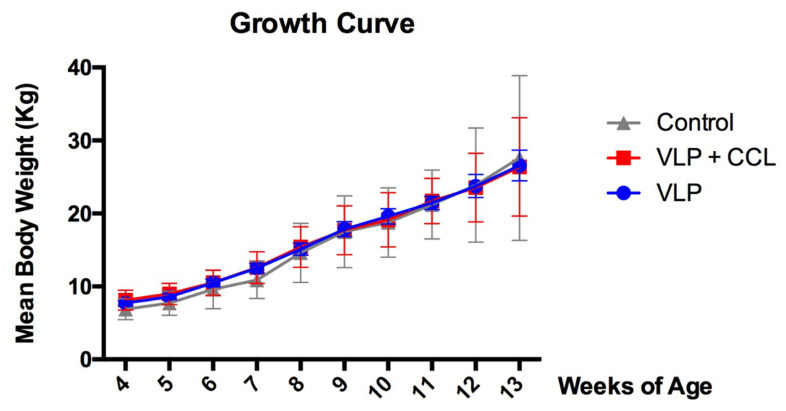
Changes of the weekly body weight. The body weights of all pigs in each group were measured every week. The weekly averaged body weights in each group are demonstrated as mean ± SEM. The mean values in control, VLP, and VLP+CCL groups are presented as gray, blue, and red lines, respectively.

The BW of each pig was measured weekly during the experiment. The BW showed a linear increase in all the groups. However, there was no significant difference in the mean BW among the three groups (Figure 6).

### 3.4. Detection of Systemic and Mucosal S-Specific Antibody Titers

**Figure 7:**
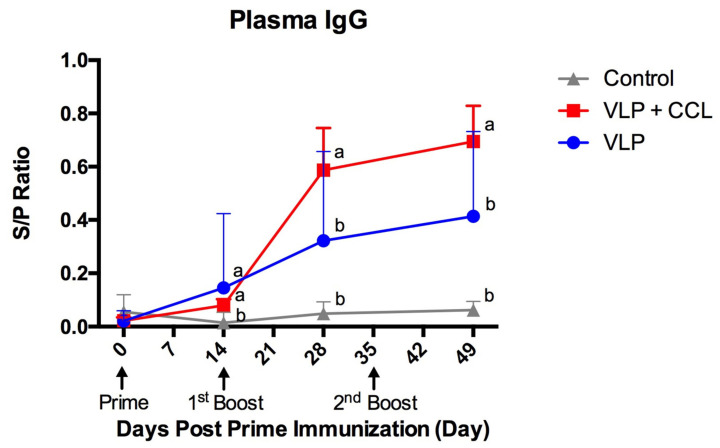
Systemic PEDV S-specific IgG titers following the prime, 1st boost, and 2nd boost. Pigs were immunized with different regimens at 0, 14, and 35 DPPI. Blood was collected at 0 (pre-priming), 14, 28, and 49 DPPI for evaluating PEDV-specific IgG titer by the PEDV S-based ELISA. The results are shown as averaged values of sample-to-positive ratios (S/P ratio) with error bars representing the SEM. The values in control, VLP, and VLP+CCL groups are demonstrated by gray, blue, and red lines, respectively. Different alphabets indicate significant differences among different groups (*p* < 0.05).

Compared to the control group, elevated IgG titers in the plasma were observed in both the VLP and VLP+CCL groups post-immunization. At 49 DPPI, the titers of control, VLP, and VLP+CCL groups were 0.06 ± 0.01, 0.41 ± 0.12, and 0.69 ± 0.13, respectively (Figure 7). The statistical method of GEE was used to assess the systemic IgG and revealed the main effects of time (Wald chi-square = 42.504, *p* < 0.001), treatment (Wald chi-square = 116.400, *p* < 0.001), and BW (Wald chi-square = 5.896, *p* = 0.015). Significant interactions of treatment × time (Wald chi-square = 724.532, *p* < 0.001), treatment × BW (Wald chi-square = 27.901, *p* < 0.001), time × BW (Wald chi-square = 16.578, *p* = 0.001), and treatment × time × BW (Wald chi-square = 136.937, *p* < 0.001) were presented. After the interaction effects of BW were removed, post hoc comparisons of the simple main effects revealed that systemic IgG levels were significantly higher in the VLP and VLP+CCL groups than in the control group at 14 DPPI, and significantly higher in the VLP+CCL group than in the control and VLP groups at 28 and 49 DPPI.

**Figure 8:**
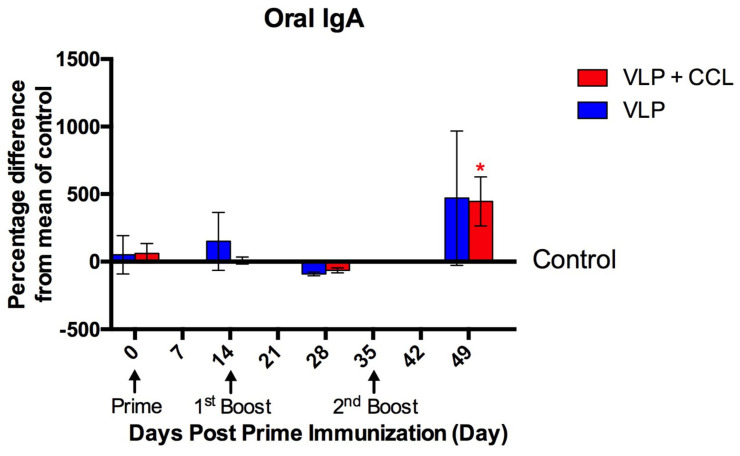
Oral PEDV S-specific IgA titers after the prime, 1st boost, and 2nd boost immunizations. Pigs were immunized at 0, 14, and 35 DPPI. Oral swabs were collected at day 0, 14, 28, and 49 DPPI to evaluate PEDV-specific IgA in the saliva by the PEDV S-based ELISA. The data are displayed as the percentage difference from the mean of control, which is defined as the percentage difference between treatment and control group divided by the mean of the control group, with SEM at different time points. The results of control, VLP, and VLP+CCL groups are presented as gray, blue, and red bars, respectively. The asterisk indicates the significant statistical difference between treatment and control groups (*p* < 0.05).

Compared to the control group, the oral IgA S/P titers elicited in the VLP and VLP+CCL groups at 49 DPPI were 0.16 ± 0.05 and 0.15 ± 0.05, respectively (Figure 8). Statistical analysis of oral IgA revealed the main effects of time (Wald chi-square = 21.983, *p* < 0.001), treatment (Wald chi-square = 11.703, *p* = 0.020), and BW (Wald chi-square = 5.674, *p* = 0.017). Significant interactions of treatment × time (Wald chi-square = 297.607, *p* < 0.001), treatment x BW (Wald chi-square = 19.334, *p* = 0.001), time × BW (Wald chi-square = 19.783, *p* = 0.001), and treatment × time × BW (Wald chi-square = 157.940, *p* < 0.001) were presented. Following the removal of the interaction effects of BW, post hoc comparisons of the simple main effects indicated that the mucosal IgA levels were significantly higher in the VLP+CCL group than in the control group at 49 DPPI.

### 3.5. Evaluation of Neutralizing Antibody Titers in the Blood

**Figure 9:**
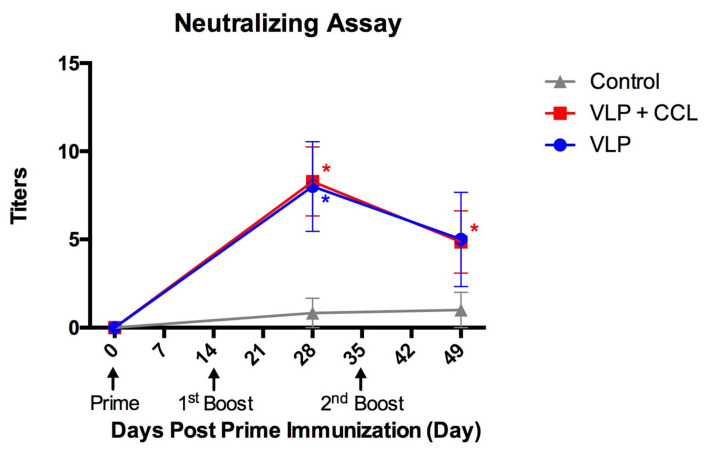
The titers of plasma neutralizing antibodies against PEDV following the prime, 1st boost, and 2nd boost immunization. The neutralizing activity against PEDVPT-P5 was performed. The values are displayed as mean ± SEM and presented as gray, blue, and red lines of control, VLP, and VLP+CCL groups, respectively. The asterisk indicates the significant statistical difference between treatment and control groups (*p* < 0.05).

Titers of neutralizing antibodies in the blood were elevated in both the VLP and VLP+CCL groups at 28 DPPI (mean ± SEM) but slightly decreased at 49 DPPI (mean ± SEM) (Figure 9). The main effects of time (Wald chi-square = 6.073, *p* = 0.048), treatment (Wald chi-square = 13.107, *p* = 0.001), and BW (Wald chi-square = 1.100, *p* = 0.294) were identified using statistical analysis. Interactions of treatment × time (Wald chi-square = 28.809, *p* < 0.001), treatment × BW (Wald chi-square = 8.196, *p* = 0.017), and treatment × time × BW (Wald chi-square = 21.322, *p* < 0.001) were significant, while time × BW (Wald chi-square = 0.621, *p* = 0.733) was non-significant. After the BW was adjusted, post hoc comparisons of the simple main effects revealed significant differences in the neutralizing antibody levels in the VLP and VLP+CCL groups at 28 DPPI, and in the VLP+CCL group at 49 DPPI, compared to the control group.

### 3.6. Assessment of S-Specific Interferon-γ-Secreting Cells in the PBMCs

**Figure 10:**
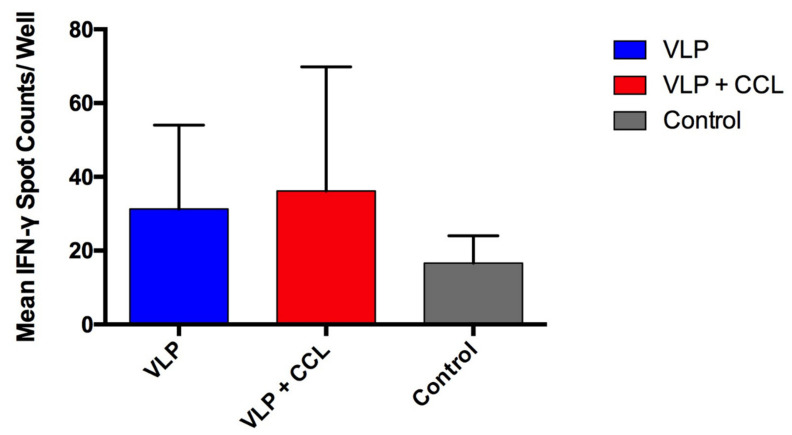
The result of PEDV S-specific Interferon-γ-secreting cell count in the peripheral blood mononuclear cells (PBMCs). The PBMCs were prepared from the peripheral blood of pigs at 49 DPPI. Interferon-γ-secreting cells were enumerated by the ELISPOT assay. The data are shown as mean ± SEM and the results of control, VLP, and VLP+CCL groups are, respectively, illustrated as gray, blue, and red bars.

To evaluate the specific cellular immunity against PEDV, we quantified the endpoint PEDV-S specific IFN-γ-secreting T-cells in PBMCs using the ELISPOT assay. Although the mean values of the VLP and VLP+CCL groups were 31.29 ± 8.59 and 36.14 ± 12.72 spot counts per well, which were higher than those in the control group (16.60 ± 7.44 spot counts per well) (Figure 10), there was no significant difference among the different groups.

### 3.7. Evaluation of the Protection Provided by the VLP Adjuvant with/without CCL25 and CCL28 against Virulent PEDV Challenge

**Figure 11:**
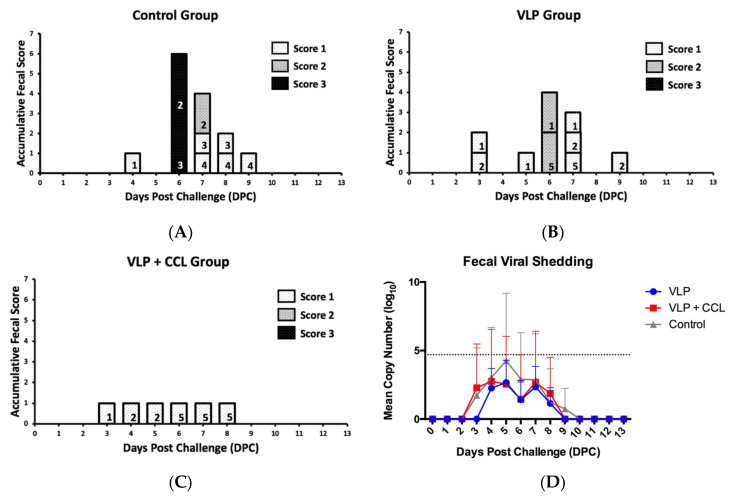
Evaluation of the protective efficacy of different treatments. Pigs in all groups were challenged by the highly virulent porcine epidemic diarrhea virus Pintung 52 (PEDV-PT) strain passage 7. The post-challenge pigs were monitored for stool consistency and fecal viral loads for 13 days. (**A**–**C**) The result of stool consistency scoring in each group. According to the stool consistency, the stool was graded as 0 for normal; 1 for loose feces; 2 for semi-fluid feces; and 3 for watery feces. The total number of pigs in the control, VLP, and VLP + CCL groups was 4, 5, and 5 pigs, respectively. The Arabic numerals labeled in the bar indicate the individuals in each group. (**D**) The result of fecal viral shedding in each group detected by probe-based quantitative reverse transcription PCR (RT-qPCR) targeting the PEDV N gene. The results were presented as mean value of log_10_ RNA copies/mL ± SEM. The detection limit of RT-qPCR was 4.7 log_10_ (copies/mL) marked as a dotted line.

To evaluate the protection offered by the different regimens, all the pigs were orally challenged with PEDVPT-P7. The onset time of diarrhea in the pigs was variable and ranged from three to six days post-challenge (DPC). Upon determination of the peak fecal score in the control group (six DPC, *n* = 4), two pigs showed watery diarrhea (score 3) but the other two pigs presented normal feces (score 0). Comparatively, when the peak fecal score was determined in the VLP group (six DPC, *n* = 5), moderate diarrhea (score 2) was observed in two pigs, mild diarrhea (score 1) in one pig, and normal feces (score 0) in two pigs, while only three pigs in the VLP+CCL group (*n* = 5) showed disconnected mild diarrhea (score 1) over three–eight DPC. The total scores of the control and VLP groups gradually decreased over six–nine DPC and no clinical signs were observed in any of the groups over 10–13 DPC. Overall, pigs immunized with VLP and VLP+CCL showed milder diarrhea symptoms than those in the control group (Figure 11A).

For quantification of PEDV loads in the feces, a PEDV N-based real-time RT-PCR was performed. In the control group, viral shedding started with 1.73 ± 3.46 log_10_ copies/mL at three DPC, reached the peak of 4.26 ± 4.92 log_10_ copies/mL at five DPC, and then declined after six DPC. In the VLP group, viral shedding was detected as 2.27 ± 3.18 log_10_ copies/mL at four DPC and fluctuated over four–eight DPC with a peak of 2.66 ± 3.65 log_10_ copies/mL at five DPC. Pigs in the VLP+CCL group exhibited average fecal viral shedding of 2.28 ± 3.20 log_10_ copies/mL at three DPC and lasted for six days with a peak of 2.75 ± 3.79 log_10_ copies/mL at four DPC (Figure 11B). Statistical analysis revealed the main effects of time (Wald chi-square = 225.571, *p* < 0.001), treatment (Wald chi-square = 10.095, *p* = 0.039), and BW (Wald chi-square = 0.097, *p* = 0.755). Significant interactions of treatment × time (Wald chi-square = 6.345 × 10^11^, *p* < 0.001), treatment × BW (Wald chi-square = 11.397, *p* = 0.022), time × BW (Wald chi-square = 224.419, *p* < 0.001), and treatment × time × BW (Wald chi-square = 55716955.8, *p* < 0.001) were presented. After the covariates of BW values in the model were fixed, although there were no significant differences in the viral shedding among all the groups, the pigs in the control group exhibited higher peak viral shedding than the other two groups.

## 4. Discussion

In this study, PEDV VLPs were generated and characterized for developing a safe and potent immunogen against highly virulent strains in pigs. To induce effective protection via a convenient parenteral route, we incorporated CCL25 and CCL28, which have been shown to be effective in our previous study [46], as mucosal adjuvants in this strategy. Our results demonstrated that the regimen is capable of eliciting not only systemic PEDV S-specific IgG and IFN-γ-producing cells in PBMCs but also mucosal PEDV S-specific IgA. Compared to the control group, the clinical signs in pigs of both the VLP and VLP+CCL groups were markedly palliated accompanied by lower viral shedding without watery diarrhea. Therefore, PEDV VLP formulated with CCL25 and CCL28 may be a potential PEDV vaccine candidate and the strategy might serve as a platform for the development of other enteric viral vaccines.

In this study, the S gene in the P-BEVS vector was in the same ORF as the M protein flanking with the 2A-like peptide sequence derived from PnV viruses [58]. Thus, the S and M proteins were translated by the same ribosome with the same yield. The E protein was controlled by the RhPV IRES, which would mediate the cap-independent translation through the same mRNA carrying the coding sequences of S and M proteins. Thus, the VLP of PEDV generated by the P-BEVS system should express the S, M, and E proteins simultaneously in the SME-Bac-infected Sf21 cells. In the present study, the detection of S, M, and E proteins by using PEDV-challenged porcine serum was performed and only the S protein was successfully detected in the SME-Bac VLP. We speculate that the failure to detect the M and E proteins using PEDV hyperimmune porcine serum by Western blotting might be due to two possibilities. First, the E and M proteins in VLPs derived from the P-BEVS system may not produce glycoproteins to generate complex M and E proteins, as the insect cell-produced glycoproteins have clearly different N-glycans from those produced by mammalian cells [59,60]. The protein structure and immunogenicity of the E and M proteins of the SME-Bac VLP might exhibit some differences from those of PEDV virions. Second, poor immunogenicity of the PEDV E protein has also been previously demonstrated [61]. Due to these detection limits, the Western blot and IFA were performed to confirm the expression of the S protein and TEM was performed to demonstrate the formation of the VLPs.

Humoral and cellular immunity play an indispensable role in the generation of an effective vaccine [62]. It is well known that lactogenic passive immune-transferring pathogen-specific IgA is one of the effective strategies to protect newborn piglets, which have immature immune systems, against G2 PEDV infection [63]. However, the crucial role of memory T cell responses in PEDV has also been proposed to protect pigs from reinfection by displaying undetected fecal viral shedding and absence of systemic and mucosal antibody responses [64]. Therefore, vaccines that can elicit both humoral and cellular immune responses might be potent in preventing the disease. Herein, after three intramuscular injections, serological and IFN-γ-secreting cell measurements revealed that the VLP and VPL+CCL groups were able to stimulate both immune responses in pigs. Both humoral and cellular immunities are measured by the interaction with in-house recombinant S protein, which is well-established regarding the confirmations of its biological function and immunogenicity in our previous studies [21,22,51,65,66] and the result could more correlate with the clinical protectivity than measured by the interaction with inactivated virions. The successful induction of both humoral and cellular immunities in the condition of using a lower amount of S protein (0.2 µg/dose) in our VLP regimen than that in other subunit vaccine studies [21,67,68] suggests that VLP is an effective strategy to induce potent humoral and cellular immune responses [69,70,71].

Secretory IgA, which is the first line of mucosal immunity, was observed to be significantly elevated after the second boost in the VLP+CCL group as compared to the control group. The statistical result seems contradictory to the raw data, which represented similar mean IgA S/P ratios between the VLP and VLP+CCL groups at 49 DPPI. Such a contradiction in the results could have arisen depending on whether the effects of BW are taken into consideration or not. The observation of enhanced IgG, IgA, and neutralizing titers in the presence of CCL25 and CCL28 is comparable to many related published reports [42,44,45,72,73] and serves as evidence that CCL25 and CCL28 are involved in chemotaxis and immunostimulation [74,75]. In addition, a milder clinical sign was also observed in pigs of the VLP+CCL group as compared to the VLP group. However, when compared to the animals immunized using the inactivated virus formulated with CCL25/28 in a previous study [46], pigs in the VLP+CCL group showed relatively less protection against the PEDV challenge. It might be due to the PEDV exposure age at 11 weeks old and failure to elicit the optimal immune response, as it can be observed that the mean PEDV S-specific IgG titers were relatively low, with mean S/P ratios of around 0.4 and 0.7 in the VLP and VLP-CCL groups, respectively. However, based on the results of our previous work, the titer of systemic IgG or neutralizing antibodies might play a minor role in the protection against PEDV [21]. In the context of the pigs injected with the same dose of CCL25/28 as in the previous study [46], the fair effect of the immunization might be caused by the suboptimal antigen concentration in the VLPs. On the other hand, the use of the soluble chemokines may also contribute to the ineffective stimulation of immune responses. Several studies have indicated that chemokine-incorporated VLPs can stimulate robust antigen-specific immune responses, while modest immune responses are noted in VLPs with soluble chemokines [76,77], highlighting the influence of co-delivery of an antigen and chemokine on promoting effective immune stimulation. Hence, the adequate regimens of VLP and CCL or even co-delivery of all the components still need further optimization and should also be applied to sows to evaluate the protection for litters via lactogenic immunity.

The immune responses elicited by immunization are affected by multiple factors, such as intrinsic host factors, perinatal host factors, and nutritional factors [78]. To evaluate the immunogenicity and potential protectivity of the novel VLP immunogen, an appropriate animal model is important for the preclinical investigation. In the present study, PEDV-seronegative post-weaning pigs were used for the VLP immunization and viral challenging experiments. This animal model has been well-established in our previous studies for preliminarily evaluating the immunogenicity of potential PEDV vaccine candidates [21,46]. After confirming the VLP regimen is capable of eliciting PEDV-specific humoral and cellular responses, considering that vulnerable suckling piglets should be protected by colostrum antibody transferred from immunized sows [63], the VLP in combination with chemokines strategy should be applied to gilts and sows to evaluate the immunogenicity and protective efficacy of lactogenic immunity in neonatal piglets against PEDVs.

To induce immunity and protectivity in pigs against the emerging G2b PEDV strains, we have successfully generated the G2b PEDV-based VLP vaccine and demonstrated the efficacy against a homologous G2b PEDV challenge in pigs. It has been reported that memory CD4^+^ T cells against human cold coronaviruses, such as human coronavirus (HCoV) OC43, HCoV-229E, HCoV-NL63, and HCoV-HKU1, and monoclonal IgA against severe acute respiratory syndrome coronavirus (SARS-CoV) can provide cross-reactivity against severe acute respiratory syndrome coronavirus 2 (SARS-CoV-2) [79,80]. As for the PEDVs, the nucleotide sequences of the S protein among G1 and G2 strains differ within 10%, and antiserum of G2 PEDVs has been proved to be able to provide partial cross-reactivity against G1 PEDVs and vice versa [15,81,82]. Accordingly, the vaccines derived from G2 strains might cross-protect pigs against G1 PEDV infection. The efficacy of our G2b-based PEDV VLP vaccine against G1 PEDVs should also be evaluated in the future.

In the present study, although the regimen used for immunization of the VLP+CCL group still needs to be modified, the strategy was able to induce mucosal and systemic immune responses via intramuscular administration. In addition, it also provided partial protection by resulting in palliated clinical signs and reduced viral shedding following challenge with the highly virulent PEDV strain. Additionally, VLP derived from P-BEVS serves as a potent immunogen that is capable of inducing humoral and cellular immunities in pigs. Of note, this study also points out the importance of integrating BW as a covariate into evaluation when investigating vaccine efficacy. In summary, VLP in combination with CC chemokines could be a promising candidate for mucosal vaccines against other enteric or mucosal pathogens.

## Figures and Tables

**Table 1:** Vaccine formulation and immunization program.

Group	Immunogen	Adjuvant	
		CC Chemokine	Freund’s Adjuvant *
Control	None	None	Yes
VLP	1.8 mg of VLP (0.2 µg S protein)	None	Yes
VLP + CCL25/28	1.8 mg of VLP (0.2 µg S protein)	30 µg CCL25 and 30 µg CCL28	Yes

* 1st immunization: 0.5 mL of complete Freund’s adjuvant; 2nd immunization: 0.5 mL of incomplete Freund’s adjuvant; 3rd immunization: 0.5 mL of incomplete Freund’s adjuvant.
